# Interaction between Endothelial Protein C Receptor and Intercellular Adhesion Molecule 1 to Mediate Binding of *Plasmodium falciparum*-Infected Erythrocytes to Endothelial Cells

**DOI:** 10.1128/mBio.00615-16

**Published:** 2016-07-12

**Authors:** Marion Avril, Maria Bernabeu, Maxwell Benjamin, Andrew Jay Brazier, Joseph D. Smith

**Affiliations:** a Center for Infectious Disease Research (formerly Seattle Biomedical Research Institute), Seattle, Washington, USA; b Department of Global Health, University of Washington, Seattle, Washington, USA

## Abstract

Intercellular adhesion molecule 1 (ICAM-1) and the endothelial protein C receptor (EPCR) are candidate receptors for the deadly complication cerebral malaria. However, it remains unclear if *Plasmodium falciparum* parasites with dual binding specificity are involved in cytoadhesion or different parasite subpopulations bind in brain microvessels. Here, we investigated this issue by studying different subtypes of ICAM-1-binding parasite lines. We show that two parasite lines expressing domain cassette 13 (DC13) of the *P. falciparum* erythrocyte membrane protein 1 (PfEMP1) family have dual binding specificity for EPCR and ICAM-1 and further mapped ICAM-1 binding to the first DBLβ domain following the PfEMP1 head structure in both proteins. As PfEMP1 head structures have diverged between group A (EPCR binders) and groups B and C (CD36 binders), we also investigated how ICAM-1-binding parasites with different coreceptor binding traits influence *P. falciparum*-infected erythrocyte binding to endothelial cells. Whereas levels of binding to tumor necrosis factor alpha (TNF-α)-stimulated endothelial cells from the lung and brain by all ICAM-1-binding parasite lines increased, group A (EPCR and ICAM-1) was less dependent than group B (CD36 and ICAM-1) on ICAM-1 upregulation. Furthermore, both group A DC13 parasite lines had higher binding levels to brain endothelial cells (a microvascular niche with limited CD36 expression). This study shows that ICAM-1 is a coreceptor for a subset of EPCR-binding parasites and provides the first evidence of how EPCR and ICAM-1 interact to mediate parasite binding to both resting and TNF-α-activated primary brain and lung endothelial cells.

## IMPORTANCE

Cerebral malaria is a severe neurological complication of *P. falciparum* infection associated with infected erythrocyte (IE) binding in cerebral vessels. Yet little is known about the mechanisms by which parasites adhere in the brain or other microvascular sites. Here, we studied parasite lines expressing group A DC13-containing PfEMP1 variants, a subset that has previously been shown to have high brain cell- and other endothelial cell-binding activities. We show that DC13-containing PfEMP1 variants have dual EPCR- and ICAM-1-binding activities and that both receptors are involved in parasite adherence to lung and brain endothelial cells. As both EPCR and ICAM-1 are implicated in cerebral malaria, these findings suggest the possibility that parasites with dual binding activities are involved in parasite sequestration to microvascular beds with low CD36 expression, such as the brain, and we urge more research into the multiadhesive properties of PfEMP1 variants.

## INTRODUCTION

Cerebral malaria is a life-threatening complication associated with extensive sequestration of *Plasmodium falciparum*-infected erythrocytes (IEs) in cerebral blood vessels (1, 2), but the host receptor interactions supporting cerebral binding are incompletely understood. There are numerous receptors for *P. falciparum* cytoadhesion, including CD36, intercellular adhesion molecule 1 (ICAM-1), and the endothelial protein C receptor (EPCR) (3). Recent evidence suggests that EPCR, a receptor involved in the regulation of blood clotting, inflammation, and endothelial barrier properties (4), may play a role in cerebral binding (5). EPCR-binding parasites have high cultured human brain microvascular endothelial cell-binding activity (6, 7) and are increased in severe malaria cases in children and adults (5, 8–10). In addition, ICAM-1 has been proposed to be an important receptor for cerebral binding. In autopsy studies, cerebral sequestered IEs colocalize to ICAM-1-positive vessels (11) and vessels with higher ICAM-1 levels have greater burdens of sequestered IEs (12). However, it remains unclear whether cerebral sequestered IEs have dual EPCR- and ICAM-1-binding activities or if different parasite subpopulations are involved in cerebral binding.

IE binding is mediated by specific interactions between members of the clonally variant *var* gene/*P. falciparum* erythrocyte membrane protein 1 (PfEMP1) family and receptors on the host vascular endothelium (13). PfEMP1 variants are classified into three main groups, A, B, and C, based on the upstream sequence (UpsA, UpsB, UpsC) and chromosome location (14). The PfEMP1 extracellular region contains Duffy binding-like (DBL) and cysteine-rich interdomain region (CIDR) adhesion domains, which are classified into different types (α to ζ) based on sequence similarity (15, 16). Nearly all PfEMP1 proteins contain a tandem DBL-CIDR domain at the N terminus, termed the semiconserved PfEMP1 head structure. The head structure has a major role in parasite binding specificity and has diversified between *var* groups (reviewed in reference 17). Whereas CD36 binding is the most common adhesion property of PfEMP1 variants (~84%) and is restricted to group B and C head structures (CIDRα2 to CIDRα6 domains), EPCR binding is restricted to group A head structures containing CIDRα1 domains (~11%) (5, 18, 19). A subset of PfEMP1 proteins contains the ICAM-1-binding property. This trait can be associated with either type of PfEMP1 head structure. It is associated with DBLβ5-type domains present in group B and C PfEMP1 variants (20) and with DBLβ3- and DBLβ1-type domains present in some group A PfEMP1 variants (21, 22). Given the low or absent expression of CD36 in brain microvessels (11), it is important to understand how functional diversification of ICAM-1-binding variants with either CD36- or EPCR-binding head structures may influence parasite microvascular tropism.

In this study, we isolated highly monoclonal parasite lines expressing group A DC13 PfEMP1 variants previously shown to have high human brain and other endothelial cell type binding activity (7, 23) and characterized their EPCR- and ICAM-1-binding activities. Additionally, we investigated how functional diversification of ICAM-1-binding parasite lines with CD36- or EPCR-binding head structures influences parasite binding to resting or tumor necrosis factor alpha (TNF-α)-activated microvascular endothelial cells from the lung or brain.

## RESULTS

### Derivation and characterization of parasite lines.

**FIG 1:**
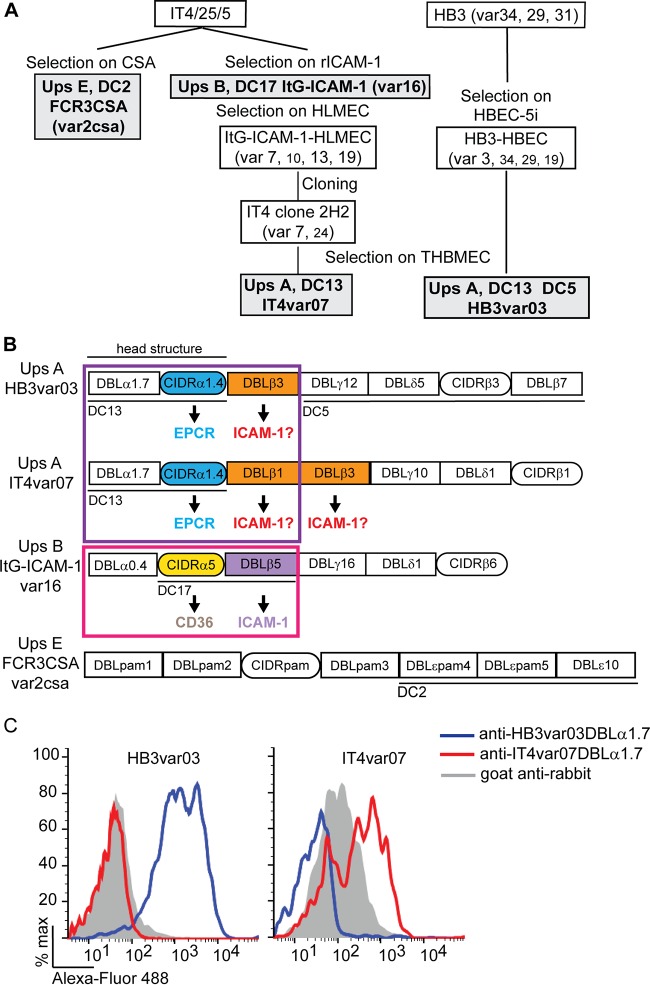
Overview of parasite selection. (A) The IT4var07 parasite line was generated from ItG−ICAM-1 by selection on HLMEC (lung cells) (23), followed by limited-dilution cloning and further selection on THBMEC, an immortalized brain endothelial cell line. The HB3var03 parasite line was originally selected on an immortalized brain endothelial cell line (HBEC-5i) (7), followed by further selection on THBMEC. The major transcribed *var* genes expressed by each parasite line are shown in a large font, and minor *var* transcripts are shown in a smaller font. The four parasite lines analyzed in this study are highlighted by gray-shaded boxes and bold lettering. (B) The extracellular domain architectures of the four PfEMP1 variants with domain cassettes underlined. Mapped receptor-binding properties are indicated by arrows, and potential ICAM-1-binding DBLβ domains are indicated by question marks. (C) Quantification of DC13 PfEMP1 expression in HB3var03- and IT4var07-IEs by flow cytometry.

To investigate the roles of EPCR and ICAM-1 in *P. falciparum* adhesion, we selected two parasite lines expressing DC13 PfEMP1 variants. The IT4var07 parasite line was originally selected on human lung endothelial cells, and the HB3var03 parasite line was originally selected on an immortalized human brain endothelial cell line (Fig. 1A). Both parent lines expressed a dominant DC13-containing *var* transcript and other *var* transcripts, including ones that encode CD36- and ICAM-1-binding activities (*IT4var13*) or are predicted to bind CD36 and ICAM-1 (*HB3var34*) (7, 23). To enrich for purer DC13 *var*-expressing parasite lines, IT4var07 was isolated by limited-dilution cloning and IT4var07 and HB3var03 parasite lines were selected by repeated panning on a transformed human brain microvascular endothelial cell (THBMEC) line. As controls we used a previously derived ICAM-1 and CD36 binder (ItG−ICAM-1, expressing *IT4var16*) and a chondroitin sulfate A (CSA)-binding parasite line (FCR3CSA, expressing *IT4var04/var2CSA*) (Fig. 1A) (24, 25).

DC13 PfEMP1 expression was confirmed by flow cytometry and *var* transcriptional profiling. By flow cytometry, nearly the entire HB3var03 parasite line was labeled by specific anti-PfEMP1 sera, and in IT4var07, there was a shift of approximately two-thirds of the parasite population (Fig. 1C). Conversely, there was no antibody cross-reactivity on the two DC13 parasite lines. Furthermore, *IT4var07* was the dominant *var* transcript in the IT4var07 parasite line by quantitative reverse transcription (qRT)-PCR with a set of primers specific to the IT4 parasite *var* gene repertoire, and *IT4var16* and *IT4var04/var2csa* were the dominant transcripts in the ItG−ICAM-1 and FCR3CSA parasite lines (see Fig. S1 in the supplemental material). As an alternative approach to *var* typing, a set of 40 domain-specific *var* primers was employed (9). As expected, none of the primers recognized the highly distinctive *var2csa* transcript expressed by FCR3-CSA. Furthermore, the domain-specific *var* primers primarily detected domains present in the IT4var07 and HB3var03 PfEMP1 variants (see Fig. S1). Taken together, these results suggest that all four parasite lines predominantly transcribed the expected *var* gene/PfEMP1 variant.

### DC13 PfEMP1-expressing parasite lines express dual binding activities for EPCR and ICAM-1.

**FIG 2:**
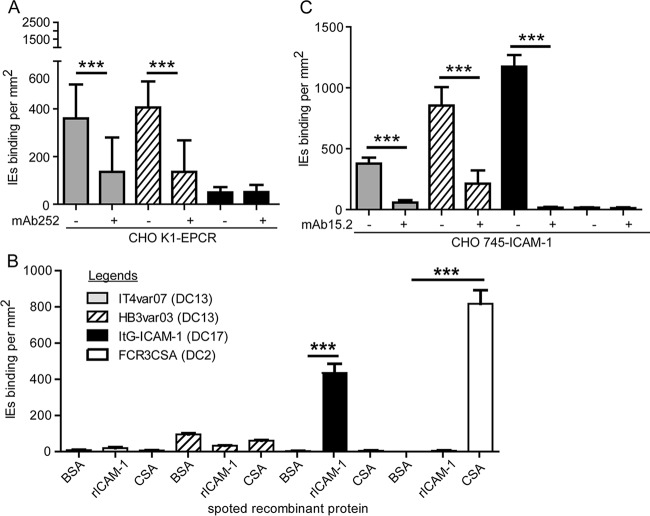
DC13 PfEMP1-expressing parasite lines exhibit dual EPCR- and ICAM-1-binding activities. (A) Binding of parasite lines to CHOK1-EPCR cells and inhibition by anti-EPCR MAb 252. The bar graphs show levels of parasite binding to CHOK1-EPCR cells after subtraction of binding to untransfected cells (CHOK1-binding levels [IEs per square millimeter]: IT4var07, 43 ± 27; HB3var03, 107 ± 40; ItG−ICAM-1, 52 ± 27 [*n* = 4 to 6 independent experiments]). (B) Binding of parasite lines to recombinant Fc−ICAM-1 protein or CSA spots. BSA spots were used as a negative control (*n* = 4 or 5 independent experiments). (C) Binding of parasite lines to CHO745−ICAM-1 cells and inhibition by anti-ICAM-1 MAb 15.2. The bar graphs show levels of parasite binding to CHO745−ICAM-1 cells after subtraction of binding to untransfected cells (CHO745-binding levels [IEs per square millimeter]: IT4var07, 120 ± 36; HB3var03, 114 ± 59; ItG−ICAM-1, 54 ± 36; FCR3CSA, 38 ± 29 [*n* = 4 or 5 independent experiments]). The bar graphs show mean values and standard errors. ***, *P* < 0.001 (Mann-Whitney test).

The DC13 cassette refers to the DBLα1.7-CIDRα1.4 tandem domain in the semiconserved head structure, but all known DC13 PfEMP1 variants contain a DBLβ domain following the head structure (Fig. 1B) (15). EPCR binding maps to the CIDRα1.4 domain (5), and previous studies have suggested that some DBLβ1 and DBLβ3 domains can bind ICAM-1 (21, 22). To investigate the binding attributes of DC13 PfEMP1-expressing parasite lines, binding assays were performed. As expected, the IT4var07 and HB3var03 parasite lines specifically bound to CHOK1-EPCR-transfected cell lines, and binding was significantly inhibited by anti-EPCR monoclonal antibody (MAb) 252 (62 and 66% reduction) (Fig. 2A). In comparison, a control ItG−ICAM-1 parasite line (CD36 and ICAM-1) showed minimal binding to CHOK1-EPCR cells.

For binding assays, both recombinant ICAM-1- and CHO−ICAM-1-transfected cell lines were employed because some parasite lines bind better to cell surface ICAM-1 than to recombinant ICAM-1 (25, 26). As expected, the control FCR3CSA (CSA-binding) and ItG−ICAM-1 (CD36- and ICAM-1-binding) parasite lines bound to CSA or recombinant ICAM-1 spots, respectively. However, neither DC13 parasite line bound to recombinant ICAM-1 (Fig. 2B). In contrast, the IT4var07, HB3var03, and positive-control ItG−ICAM-1 parasite lines specifically bound to CHO745−ICAM-1 and not CHO-745 cells. Furthermore, binding was specifically inhibited by an ICAM-1 blocking antibody, MAb 15.2 (IT4var07, 85% inhibition; HB3var03, 75% inhibition, ItG−ICAM-1, 99% inhibition) (Fig. 2C). As expected, the negative-control FCR3CSA parasite line did not adhere to CHO745−ICAM-1 cells (Fig. 2C). These data show that parasites expressing DC13-containing PfEMP1 predicted to bind EPCR and ICAM-1 can indeed bind to either receptor.

### The ICAM-1 binding site of DC13-containing PfEMP1 proteins is located in the first DBLβ domain following the PfEMP1 head structure.

**FIG 3:**
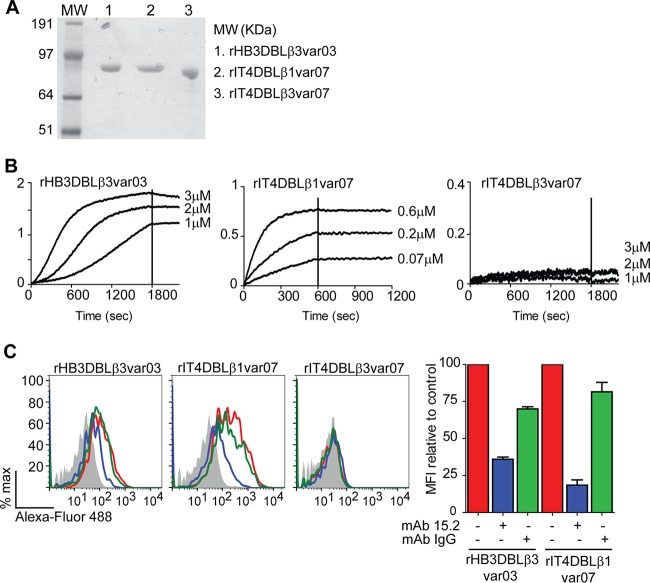
Binding specificity of DC13 DBLβ recombinant proteins for ICAM-1. (A) DBLβ1 and DBLβ3 recombinant proteins were analyzed under reducing conditions by SDS-PAGE and stained with GelCode blue protein stain. (B) Sensograms of recombinant DBLβ domain binding to recombinant Fc−ICAM-1 analyzed by BLI. (C) Binding of recombinant DBLβ domains to CHO745−ICAM-1 cells (red lines) or the negative control CHO745 parent line (gray histogram). Binding was inhibited by anti-ICAM-1 MAb 15.2 (blue lines) or an IgG isotype control MAb (green lines). A representative fluorescence-activated cell sorting histogram from two independent experiments is shown. The percent binding inhibition relative to the control is summarized in the bar graph at the right. MFI, mean fluorescence intensity.

To identify the ICAM-1-binding domain in DC13-containing PfEMP1 variants, we produced a recombinant DBLβ3 domain from HB3var03 and the DBLβ1 and DBLβ3 domains from IT4var07 PfEMP1 (Fig. 3A). Each protein ran as a single dominant band on reducing SDS-PAGE. However, the HB3var03 protein included aggregates by size exclusion chromatography, which precluded accurate measurement of binding kinetics by biolayer interferometry (BLI). Nevertheless, this methodology established that HB3var03 bound in a dose-dependent manner to the recombinant Fc−ICAM-1 probe (Fig. 3B). In addition, the first DBLβ domain in IT4var07 bound Fc−ICAM-1 with nanomolar affinity (DBLβ1; dissociation constant [*K_d_*], 8.7 nM), but the second DBLβ domain (DBLβ3) did not (Fig. 3B). Likewise, in a cell-based assay, HB3var03 DBLβ3, and IT4var07 DBLβ1 domains bound specifically to CHO745−ICAM-1 cells and not to CHO745 cells (Fig. 3C). In addition, binding was specifically inhibited by anti-ICAM-1 MAb 15.2. Conversely, the IT4var07 DBLβ3 recombinant domain did not bind CHO745−ICAM-1 cells (Fig. 3C). Taken together, these results suggest that ICAM-1 binding maps to the DBLβ domain immediately following the PfEMP1 head structure in these two DC13 PfEMP1 variants (Fig. 1B), irrespective of whether it is a DBLβ1 or a DBLβ3 domain.

### DC13 PfEMP1-expressing parasite lines adhere to EPCR and ICAM-1 on lung endothelial cells.

**FIG 4:**
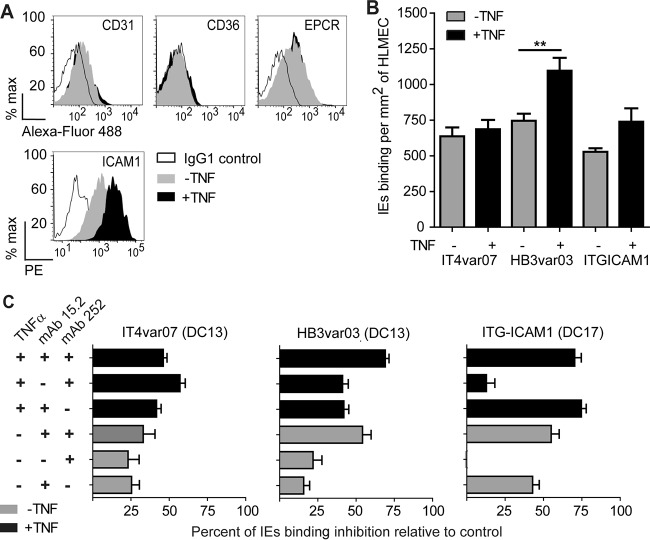
Assessment of the contributions of EPCR and ICAM-1 to parasite binding to HLMEC. (A) Levels of surface expression of CD31, CD36, ICAM-1, and EPCR on resting lung endothelial cells or after 24 h of stimulation with TNF-α. A representative experiment with human lung endothelial cells from one of two different sources is shown. PE, phycoerythrin. (B) Levels of parasite binding to resting lung endothelial cells or after 24 h of stimulation with TNF-α. (C) Percent inhibition of binding to resting or TNF-α-stimulated lung endothelial cells by anti-ICAM-1 (mAb15.2) or anti-EPCR (MAb 252) antibodies alone or in combination. Percent inhibition is based on parasite binding levels in the absence of antibody treatment. The bar graphs show mean values and standard errors (*n* = 3 to 5 independent experiments). **, *P* value of 0.001 to 0.01 (one-way ANOVA with Tukey’s posttest).

Given the microvascular heterogeneity in CD36, EPCR, and ICAM-1 expression, we compared the adhesion of ICAM-1-binding parasite lines encoding CD36- or EPCR-binding head structures to resting or TNF-α-activated lung endothelial cells. Primary human lung microvascular endothelial cells (HLMECs) grew well initially but generally stopped dividing by passage 5 or 6. Resting primary human microvascular lung endothelial cells expressed the constitutive endothelial marker CD31, as well as EPCR and ICAM-1, but had low surface CD36 expression (Fig. 4A). At early passages (3 to 5) used for parasite binding assays, the cells were 100% positive for CD31 expression by immunofluorescence assay and approximately 30% were CD36 surface positive (23). However, by later passages (5 and 6), necessary for flow cytometry, only a subpopulation of cells was CD31 positive and few cells were CD36 positive (Fig. 4A). Overnight TNF-α activation led to upregulation of ICAM-1 in the majority of the cells but did not change surface CD36 and EPCR levels (Fig. 4A).

On resting lung endothelial cells, the two DC13-expressing parasite lines IT4var07 and HB3var03 (EPCR and ICAM-1) had binding activity similar to that of ItG−ICAM-1 (CD36 and ICAM-1 binder) (637 ± 62, 746 ± 49, and 528 ± 25 IEs/mm^2^, respectively) (Fig. 4B). Levels of binding to TNF-α-stimulated lung endothelial cells increased 1.5-fold for the HB3var03 parasite line and 1.4-fold for the ItG−ICAM-1 parasite line, while the binding of IT4var07 was unchanged (Fig. 4B).

To examine the roles of EPCR and ICAM-1 in parasite adhesion, blocking antibodies were employed. While anti-EPCR MAb 252 slightly reduced the binding of both DC13 parasite lines to resting lung endothelial cells (HB3var03, 22% inhibition; IT4var07, 23% inhibition; gray bars in Fig. 4C), the effect was increased on TNF-α-stimulated cells (HB3var03, 41% inhibition; IT4var07, 57% inhibition). As expected, anti-EPCR MAb 252 did not inhibit the control ItG−ICAM-1 parasite line (Fig. 4C). In comparison, the ItG−ICAM-1 parasite line was more sensitive to ICAM-1 blockade on either resting or TNF-α-stimulated lung endothelial cells (43 and 75% inhibition) than IT4var07 (25 and 42% inhibition) and HB3var03 (16 and 42% inhibition) were (Fig. 4C). The finding that ICAM-1 blockade was greater for all three parasite lines on TNF-α-stimulated lung endothelial cells suggests that the upregulation of ICAM-1 was at least partly responsible for the increased parasite binding to activated lung endothelial cells.

Of interest, the combination of anti-EPCR and anti-ICAM-1 antibodies led to a further reduction of binding by the HB3var03 DC13 parasite line on both resting (54% inhibition) and TNF-α-stimulated lung endothelial cells (69% inhibition), but this was not observed for IT4var07 or the control ItG−ICAM-1 parasite line (CD36 and ICAM-1) (Fig. 4C). Therefore, although both DC13 variants bound to ICAM-1 and EPCR on lung endothelial cells, there was greater cooperation between the two receptors for the HB3var03 parasite than for IT4var07. Furthermore, there was heterogeneity among the three ICAM-1-binding parasite variants in host receptor blockade.

### DC13 PfEMP1-expressing parasite lines adhere to EPCR and ICAM-1 on brain endothelial cells.

**FIG 5:**
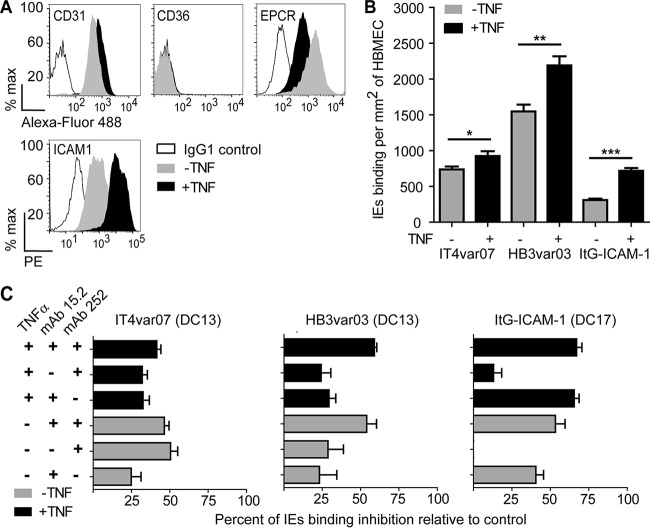
Assessment of the contributions of EPCR and ICAM-1 to parasite binding to HBMEC. (A) Levels of surface expression of CD31, CD36, ICAM-1, and EPCR on resting brain endothelial cells or after 24 h of stimulation with TNF-α. (B) Levels of parasite binding to resting brain endothelial cells or after 24 h of stimulation with TNF-α. (C) Percent inhibition of binding to resting or TNF-α-stimulated brain endothelial cells by anti-ICAM-1 (MAb 15.2) or anti-EPCR (MAb 252) antibodies alone or in combination. Percent inhibition is based on parasite binding levels in the absence of antibody treatment. The bar graphs show mean values and standard errors (*n* = 3 independent experiments). ***, *P* < 0.001; **, *P* = 0.001 to 0.01; *, *P* = 0.01 to 0.05 (one-way ANOVA with Tukey’s posttest).

CD36 is expressed at low levels on brain microvascular endothelial cells (11). To investigate how this may influence parasite tropism, we compared ICAM-1 parasite variants encoding either CD36- or EPCR-binding head structures. Primary brain endothelial cells could be grown for more passages than lung endothelial cells. Resting primary human brain microvascular endothelial cells expressed the constitutive endothelial marker CD31, as well as EPCR and ICAM-1, and were negative for CD36. Overnight TNF-α activation led to upregulation of ICAM-1 and downregulation of EPCR surface levels in brain endothelial cells (Fig. 5A), consistent with previous reports on human umbilical vein endothelial cells (27).

In contrast to lung endothelial cells, ItG−ICAM-1 (CD36 and ICAM-1 binder) showed 2- to 4-fold lower binding (308 ± 21 IEs/mm^2^) to resting brain endothelial cells than IT4var07 (736 ± 40 IEs/mm^2^) and HB3var03 (1545 ± 96 IEs/mm^2^) did (Fig. 5B). In addition, ItG−ICAM-1 was more responsive to TNF-α stimulation (2.3-fold increase) than IT4var07 and HB3var03 were (1.2- and 1.4-fold increases, respectively) (Fig. 5B). This finding suggests that the ItG−ICAM-1 parasite was more dependent on ICAM-1 for adhesion to CD36-negative brain endothelial cells than the two DC13 parasite lines were.

Consistent with this interpretation, ICAM-1 blockade had a greater effect on ItG−ICAM-1 than either DC13 parasite line on resting brain endothelial cells (IT4var07, 25% inhibition; HB3var03, 23% inhibition; ItG−ICAM-1, 41% inhibition) and on TNF-α-stimulated primary brain endothelial cells (IT4var07, 33% inhibition; HB3var03, 29% inhibition; ItG−ICAM-1, 66% inhibition) (Fig. 5C). In addition, anti-EPCR MAb 252 partially inhibited the binding of both DC13 parasite lines to resting brain cells (IT4var07, 49% inhibition; HB3var03, 29% inhibition) or TNF-α-stimulated brain cells (32 and 24% inhibition, respectively) but had no effect on that of the control ItG−ICAM-1 parasite line (Fig. 5C).

While ICAM-1 blockade alone had limited effectiveness against DC13-expressing parasite lines, the combination of anti-EPCR and anti-ICAM-1 antibodies led to a greater reduction in the binding of the HB3var03 parasite line to both resting and TNF-α-stimulated brain cells (54 and 59% inhibition, respectively) (Fig. 5C). However, similar to lung endothelial cells, there was no cooperation in antibody blockade for IT4var07 or the control ItG−ICAM-1 parasite line (Fig. 5C). Taken together, these results show that EPCR and ICAM-1 contribute to the adhesion of DC13-containing PfEMP1 variants but suggest that the relative contributions to the parasite variants differ.

## DISCUSSION

ICAM-1 and EPCR are candidate receptors for cerebral malaria, but there has been limited characterization of parasite lines with both adhesion traits. Analysis of PfEMP1 protein binding has revealed that ICAM-1 binding domains can be linked to either group B and C (CD36-binding) or group A (EPCR-binding) head structures (17). However, the functional consequence of this binding dichotomy for parasite microvascular tropism has not been explored. In this study, we investigated the microvascular specificity of different types of ICAM-1-binding parasite lines.

ICAM-1 is constitutively expressed at low levels on resting endothelial cells and is widely upregulated in different microvascular beds in malaria infection (11). ICAM-1 has been suggested to be an important receptor in cerebral binding, but the findings are inconclusive. Cerebral sequestered parasites colocalize to ICAM-1-positive microvessels (11), but the level of ICAM-1-binding parasites is not always higher in cerebral malaria cases (28–30). Whereas previous work has shown that CD36 and ICAM-1 cooperate in IE binding to dermal endothelial cells (26, 31), brain endothelial cells have limited CD36 expression, indicating that other receptors may be important for cerebral tropism. Indeed, parasites selected *in vitro* on human brain endothelial cells switched away from CD36-binding variants and had increased expression of DC8 (group B/A) and DC13 (group A) containing PfEMP1 that were subsequently found to have EPCR-binding activity (5–7). Thus, the binding specificity of the PfEMP1 head structure for CD36 or EPCR may have an important role on parasite microvascular specificity.

Despite their importance for severe malaria, relatively little is known about group A PfEMP1 variants because their expression tends to be downregulated in lab-adapted parasite lines. Group A appears to have subspecialized into rosetting variants (binding to uninfected erythrocytes) (32, 33) and EPCR-binding variants (5, 18), but the coreceptor-binding traits of EPCR binders remain unknown. In this study, we show that parasite lines expressing group A DC13-containing PfEMP1 variants have dual EPCR- and ICAM-1-binding activities and map ICAM-1 binding to the DBLβ domain immediately following the PfEMP1 head structure in IT4var07 and HB3var03. Previously, we were unable to show that IT4var07 DBLβ1 binds ICAM-1 by using a Cos-7 protein expression system (20), but we presume that this must have been a false-negative finding. Significantly, both DC13 parasite lines adhered to ICAM-1 in cell-based assays but failed to bind to recombinant ICAM-1 in protein spot assays. This has also been occasionally observed for other ICAM-1-binding parasite lines (25, 26). This disparity between assays has important practical implications because recombinant ICAM-1 spotted assays are commonly used in field studies because of their ease of application and may therefore underestimate the true proportion of ICAM-1-binding variants.

Previously, some DBLβ3 and DBLβ1 domains in group A variants have been shown to bind ICAM-1 (21, 22), but this is the first time that dual EPCR- and ICAM-1-binding parasites have been characterized for endothelial binding. On the basis of domain composition, group A PfEMP1 variants encoding either a DC4 cassette (DBLα1.4-CIDRα1.6-DBLβ3) or a DC13 cassette (DBLα1.7-CIDRα1.4, followed by DBLβ1/β3) may have dual EPCR- and ICAM-1-binding activities. It is not uncommon for group A PfEMP1 head structures to be followed by a DBLβ1 or DBLβ3 domain (15), but these sequences are relatively diverse (see Fig. S2 in the supplemental material), and it remains unclear if other group A PfEMP1 variants, besides DC4 and DC13, have dual EPCR- and ICAM-1-binding activities. Notably, some PfEMP1 variants have a tandem arrangement of DBLβ domains, but so far, ICAM-1-binding activity has only been mapped to the first DBLβ domain immediately following the head structure (20–22). It is interesting to speculate that this structural arrangement between the head structure and the ICAM-1-binding domain may be under evolutionary selection in PfEMP1 proteins to optimize binding cooperation. Future studies are needed to determine if a single PfEMP1 protein can simultaneously engage both receptors.

Although parasites expressing DC13 PfEMP1 have broad tropism for brain, lung, and cardiac endothelial cells (23), much less is known about how EPCR and ICAM-1 may interact to facilitate parasite binding. We show that DC13 parasite lines showed greater binding of resting brain endothelial cells (a microvascular niche with limited CD36 expression) than a CD36- and ICAM-1-binding parasite line. Furthermore, DC13 parasites had a fewer-fold binding increase on TNF-α-stimulated cells and were less sensitive to ICAM-1 blockade on resting and activated brain and lung endothelial cells. Significantly, a combination of EPCR and ICAM-1 antibodies was insufficient to prevent the binding of any parasite line to brain or lung endothelial cells and there was heterogeneity in receptor blockade between parasite lines, suggesting the possibility that additional receptors are involved in binding.

A limitation of this study is that binding was studied under static adhesion conditions. However, our findings are consistent with previous results showing that EPCR only partially contributes to IT4var07 parasite binding to resting brain and lung endothelial cells under flow (34). An additional limitation is that only a limited number of parasite lines and microvascular endothelial cell sources were studied, and the extent of microvascular heterogeneity within an organ or between individuals is unclear. Nevertheless, our findings and those of others (21) suggest the possibility that parasites with dual EPCR- and ICAM-1-binding activities are involved in cerebral binding. It also remains possible that some parasites bind EPCR but not ICAM-1. For instance, the EPCR-binding DC8 PfEMP1 variants are strongly selected on human brain endothelial cells *in vitro* (6, 7) and some group A PfEMP1 variants with EPCR-binding head structures lack DBLβ domains. More work is necessary to characterize the specific binding attributes of cerebral sequestered parasites. The demonstration that ICAM-1-binding parasites have diverged in EPCR and CD36 coreceptor adhesion traits has particular significance for efforts to correlate malaria clinical syndromes to specific parasite binding types. In complex binding systems, this could create “noise” when attempting to correlate single receptors with disease.

In conclusion, our study demonstrates that ICAM-1-binding variants can be grouped into CD36 and EPCR coreceptor binding traits and has important implications for understanding parasite tropism for different microvascular beds, as well as disease mechanisms.

## MATERIALS AND METHODS

### Parasite lines.

*P. falciparum* parasites were cultured under standard conditions with human type O red blood cells in RPMI 1640 medium (Invitrogen) supplemented with 10% pooled human type A positive serum. The ItG−ICAM-1 parasite line was derived by selection on purified ICAM-1 protein (35). The IT4var07 parasite line was generated by repetitive selection of the ItG−ICAM-1 parasite line on primary HLMEC (23), followed by limited-dilution cloning and one more selection on THBMECs. The FCR3CSA line (expresses *IT4var04/var2csa*) was selected on recombinant CSA (24). The HB3var03 parasite line was kindly provided by Alex Rowe (University of Edinburgh) (7) and subsequently selected on THBMECs.

### Flow cytometry assay on IEs.

Flow cytometry was done as previously described on mature-stage IEs grown in type O negative blood (24). IEs were incubated for 30 min on ice with either 0.2 µg/ml purified IgG from a rabbit immunized with NTS-IT4var07DBLα1 or 0.1 µg/ml purified IgG from a rabbit immunized with NTS-HB3var03DBLα1 (both gifts from Alex Rowe, United Kingdom). Bound antibodies were detected by adding Alexa fluor 488-conjugated goat anti-rabbit IgG (A-11034; Molecular Probes) at 1/500. Samples were analyzed in an LSRII (Becton, Dickinson) with FlowJo V10 software (Tree Star Inc.).

### Determination of *var* transcription by qRT-PCR.

The *var* gene transcription profiles of parasite lines were determined with a set of gene-specific primers to the IT4 *var* repertoire (25) or a set of PfEMP1 domain-specific primers (9). In brief, RNA was extracted from ring stage parasites at ~8 to 12 h postinvasion. Real-time reactions were performed in an ABI Prism 7500 Thermal Cycler with Power-SYBR green Master Mix and previously published PCR conditions for the respective primer sets (9, 25). Relative transcription of the IT4 *var* primer set was normalized to the control housekeeping gene for adenylosuccinate lyase (PFB0295w), and relative transcription of the domain specific primer set was normalized to the averaged transcript abundance of the control housekeeping genes for seryl-tRNA-synthetase (STS; PF07_0073) and arginyl-tRNA-synthetase (ARG; PFL0900c) as follows: Δ*C_T_* var_primer = *C_T_* var_primer − *C_T_* average_housekeeping primers. The level of *var* expression was represented as transcript units (TU) and calculated as TU = 2^5 − Δ*CT*^ as previously described (9).

### Production of recombinant PfEMP1 domains.

Recombinant PfEMP1 domains were synthesized as gBlocks gene fragments (Integrated DNA Technologies) and produced as His6-MBP-TEV-PfEMP1 insert-StrepII-tagged proteins in pSHuffle Express (NEB) expression hosts as previously described (6). The proteins made were IT4var07DBLβ1 (N726-C1211), IT4var07DBLβ3 (N1227-C1677), and HB3var03DBLβ3 (N728-C1209). Proteins were quantitated via bicinchoninic acid assay (Pierce) before visualization by reducing 4 to 12% bis-Tris SDS-PAGE (Invitrogen) and GelCode Blue staining (Thermo). Purified proteins were stored at −80°C until use.

### BLI analysis.

Binding of DBLβ domains to an Fc−ICAM-1 probe was determined on the Octet QKe instrument (ForteBio, Inc.) as described previously (36). The mean apparent *K_d_* value was determined from double-reference-subtracted data from three concentrations that were fitted globally to a 1:1 Langmuir binding model with data analysis software.

### Human microvascular endothelial cell cultures.

Two sources of primary HLMECs were used. One was purchased from ScienCell (HPMEC 3000) and cultured in accordance with the manufacture’s recommendations in a fibronectin-coated support. The other was purchased from Lonza (HMVEC-L cc-2527) and cultured in accordance with the manufacture’s recommendations in the EGM-2 MV Bulletkit (cc-3202). Both primary lung endothelial cell lines were used between passages 3 and 5 for parasite binding assays and by passage 7 for flow cytometry. Primary human brain microvascular endothelial cells were purchased from Cell Systems (acbri 376) and cultured in accordance with the manufacture’s recommendations in a collagen-coated support for 2 passages. Brain endothelial cells were cultivated up to passage 10 with the Lonza media EGM-2 MV Bulletkit and with the attachment factor (4z0-210; Cell Systems) as a growth support.

### Recombinant PfEMP1 domain binding to transfected CHO cells.

Stably transfected CHOK1-EPCR or CHO745−ICAM-1 cells or the respective untransfected control cells were lifted with 10 mM EDTA and washed with phosphate-buffered saline (PBS). A total of 1 × 10^5^ cells were incubated with 50 µg/ml recombinant DBLβ protein. Binding was detected by rabbit anti-StrepII tag polyclonal antibodies (NWSHPQFEK antibody at 1/40; GenScript), followed by goat anti-rabbit Alexa fluor 488-coupled antibodies (1/500; Molecular Probes). For inhibition assays, anti-human ICAM-1 (clone 15.2, 10 µg/ml; Abcam ab20) or isotype control IgG1 (10 µg/ml) antibodies were added 30 min prior to the addition of recombinant DBL proteins. Data were analyzed in an LSRII (Becton, Dickinson) with FlowJo v10 software (Tree Star Inc.).

### IE binding assays.

Assays of IE binding to CHO745 (CSA surface negative), CHO745−ICAM-1, and CHOK1-EPCR transfectants were performed as previously described (25). For binding inhibition assays, anti-human ICAM-1 MAb 15.2 (5 µg/ml) or a rat anti-human EPCR MAb (50 µg/ml, clone RCR-252; Sigma E6280) was added to cells for 30 min of incubation at 37°C prior to the addition of IEs. Nonbinding IEs were removed by inversion of the slide into a glass slide washing chamber. For protein spot assays, a 10-µl spot at 50 µg/ml was employed for recombinant Fc−ICAM-1 (720-IC; R&D Systems) and CSA (Sigma). Binding was quantified by determining the number of IEs adhering per square millimeter in four random fields at ×400 magnification. Bovine serum albumin (BSA) spots were used as a negative control.

For primary human brain and lung microvascular endothelial cell binding assays, cells were seeded onto coated eight-well slides (BD Biocoat) for 3 to 4 days and allowed to grow to confluence. Binding assays and washes were performed with prewarmed binding medium (RPMI 1640 medium containing 0.5% BSA, pH 6.8) as previously described (23). Briefly, IE suspensions were enriched to 30 to 60% parasitemia by gelatin enrichment. For antibody binding inhibition assays, anti-human ICAM-1 MAb 15.2 (10 µg/ml), anti-human EPCR MAb 252 (50 µg/ml), or a mixture of the two was added 30 min prior to the IE suspension. To quantify IE binding, slides were fixed in 1% glutaraldehyde and stained with 1× Giemsa. Binding was quantified by determining the number of IEs adhering per square millimeter of endothelial cells in 12 random fields at ×400 magnification. For binding assays with cytokine stimulation, confluent monolayers of endothelial cells were stimulated with 10 ng/ml TNF-α (T0157; Sigma) for 20 h at 37°C before analysis. All binding assays were scored and quantified by observers who were blind to the assay conditions.

### Flow cytometry of primary endothelial cells.

For TNF-α stimulation, confluent monolayers of primary endothelial cells were stimulated with 10 ng/ml TNF-α for 20 h at 37°C. For flow cytometry, cells were rinsed with Hanks balanced salt solution and lifted with 10 mM EDTA. EPCR was detected by a goat anti-human EPCR polyclonal antibody (30 µg/ml; R&D), followed by a fluorescently labeled secondary antibody. ICAM-1 was detected by a phycoerythrin-labeled mouse MAb (0.5 µg/ml; Abcam ab19756). CD36 was detected by a fluorescein isothiocyanate (FITC)-conjugated mouse MAb (clone TR9, 10 µl/10^5^ cells; Abcam ab39022), and CD31 was detected by an FITC-conjugated mouse MAb (clone B-B38, 1 µl/10^5^ cells; Abcam ab27333). Prior to LSRII analysis, cells were fixed with 2% (wt/vol) paraformaldehyde for 10 min. Single live cells were gated with the Live/Dead fixable violet Dead cell stain kit (Molecular Probes), and data were analyzed with FlowJo v10 software.

### Statistical analyses.

Statistical analyses were performed with Prism software. Data were analyzed by the nonparametric Mann-Whitney test or one-way analysis of variance (ANOVA) with Tukey’s posttest.

## SUPPLEMENTAL MATERIAL

Figure S1  Transcriptional profiling of *var* genes in parasite lines. (a) The transcription of *var* genes from ring stage parasites was analyzed by qRT-PCR with an IT4 parasite-specific primer set. Results are normalized to the housekeeping control gene for adenylosuccinate lyase (*asl*). Genes are organized by Ups group category; nd indicates that the Ups type has not been determined. *IT4var4* is an alternative name for *var2csa* in the FCR3CSA parasite line. (b) The transcription of *var* genes was analyzed by qRT-PCR with a set of 40 domain-specific primer pairs or a *var2CSA* gene-specific primer. The genes for STS (seryl-tRNA synthetase) and ARG (arginyl-tRNA synthetase) are housekeeping genes used to compare the *var* gene levels of parasite lines. Asterisks represent *var* genes predicted to be amplified from each parasite strain by *in silico* prediction, and gray dots represent a *var* gene present within the respective IT4/FCR3 or HB3 parasite genotype. Download Figure S1, PDF file, 0.4 MB

Figure S2  Phylogeny of DBLβ1 and DBLβ3 domains. A neighbor-joining cladogram of 38 DBLβ1/β3 domains that are located immediately after the PfEMP1 head structure is shown. PfEMP1 variants encoding EPCR-binding CIDR domain subtypes are labeled with an asterisk. Recombinant DBLβ1 or DBLβ3 domains that have been shown to bind ICAM-1 are underlined in red font. The Ups group that each gene belongs to is indicated by the letter within a box, and the types of domain cassettes present in each protein are indicated by ovals. The edge number by the origin of a branch is the bootstrap value (>80%). Download Figure S2, PDF file, 0.4 MB
