# Supplementary material for: Interaction between Endothelial Protein C Receptor and Intercellular Adhesion Molecule 1 to Mediate Binding of Plasmodium falciparum-Infected Erythrocytes to Endothelial Cells
Source: mBio. 2016 Jul 12;7(4):e00615-16. doi: 10.1128/mBio.00615-16 (PMC4958245; doi:10.1128/mBio.00615-16)
Supplement: Figure S1 — Transcriptional profiling of var genes in parasite lines. (a) The transcription of var genes from ring stage parasites was analyzed by qRT-PCR with an IT4 parasite-specific primer set. Results are normalized to the housekeeping control gene for adenylosuccinate lyase (asl). Genes are organized by Ups group category; nd indicates that the Ups type has not been determined. IT4var4 is an alternative name for var2csa in the FCR3CSA parasite line. (b) The transcription of var genes was analyzed by qRT-PCR with a set of 40 domain-specific primer pairs or a var2CSA gene-specific primer. The genes for STS (seryl-tRNA synthetase) and ARG (arginyl-tRNA synthetase) are housekeeping genes used to compare the var gene levels of parasite lines. Asterisks represent var genes predicted to be amplified from each parasite strain by in silico prediction, and gray dots represent a var gene present within the respective IT4/FCR3 or HB3 parasite genotype. Download [file mbo004162898sf1.pdf]

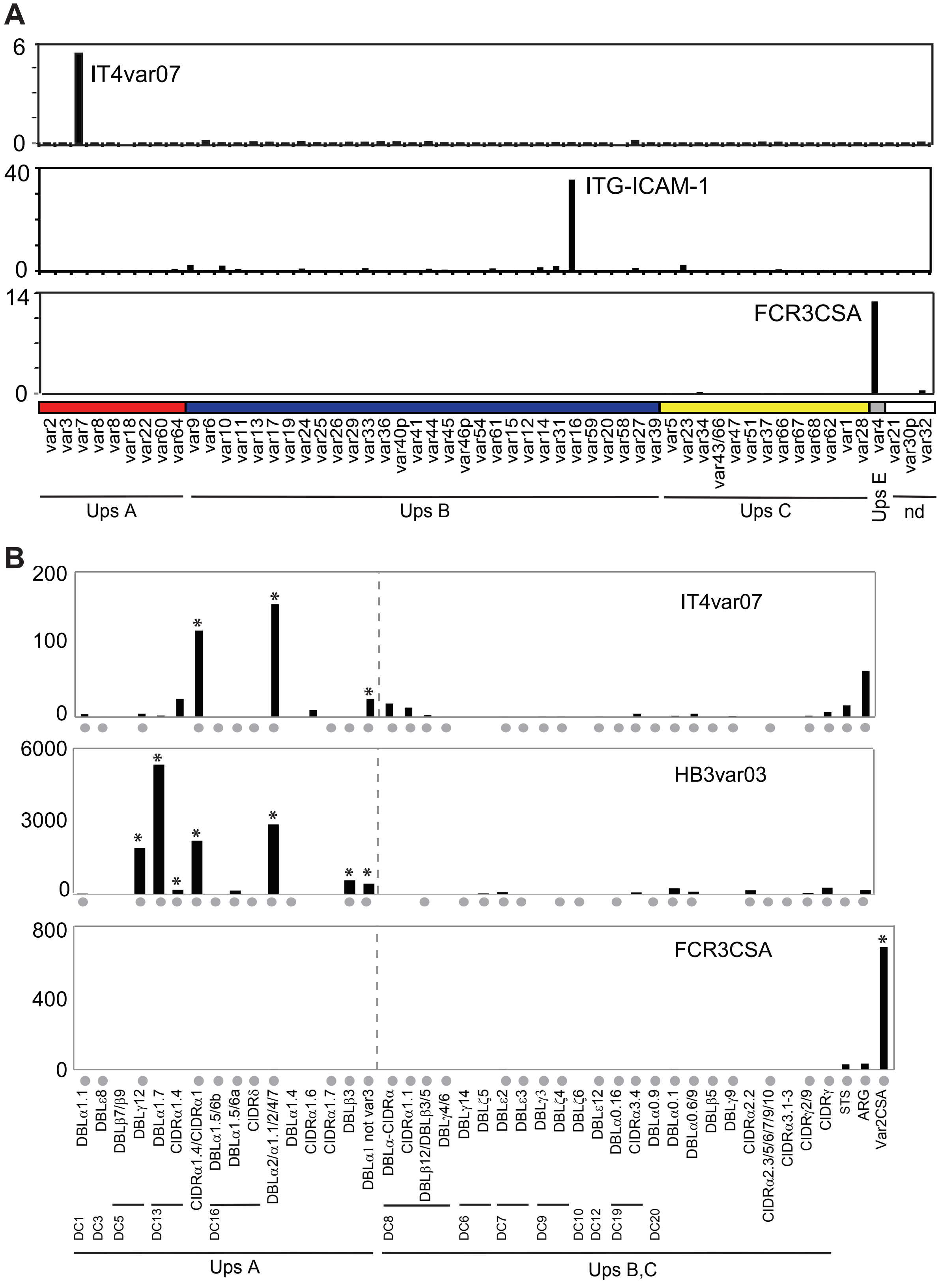

FIG. S1
Transcriptional profiling of var genes in parasite lines. (A) The transcription of var genes was analyzed by Q-RT-PCR from ring stage parasites using an IT4 parasite-specific primer set. Results are normalized to the housekeeping control gene adenylosuccinate lyase (asl). Genes are organized by Ups Group category; nd = Ups type has not been determined. IT4var4 is alternative name for var2csa in the FCR3CSA parasite line. (B) The transcription of var genes was analyzed by Q-RT-PCR using a set of 40 domain-specific primer pairs or a var2CSA gene-specific primer. STS (Seryl-tRNA synthetase) and ARG (Arginyl-tRNA synthetase) are housekeeping genes used to compare var gene levels between parasite lines. Asterisks represent var genes predicted to be amplified from each parasite strain by in silico prediction and grey dots represents a var gene present within the respective IT4/FCR3 or HB3 parasite genotypes.
