# Supplementary material for: Interaction between Endothelial Protein C Receptor and Intercellular Adhesion Molecule 1 to Mediate Binding of Plasmodium falciparum-Infected Erythrocytes to Endothelial Cells
Source: mBio. 2016 Jul 12;7(4):e00615-16. doi: 10.1128/mBio.00615-16 (PMC4958245; doi:10.1128/mBio.00615-16)
Supplement: Figure S2 — Phylogeny of DBLβ1 and DBLβ3 domains. A neighbor-joining cladogram of 38 DBLβ1/β3 domains that are located immediately after the PfEMP1 head structure is shown. PfEMP1 variants encoding EPCR-binding CIDR domain subtypes are labeled with an asterisk. Recombinant DBLβ1 or DBLβ3 domains that have been shown to bind ICAM-1 are underlined in red font. The Ups group that each gene belongs to is indicated by the letter within a box, and the types of domain cassettes present in each protein are indicated by ovals. The edge number by the origin of a branch is the bootstrap value (>80%). Download [file mbo004162898sf2.pdf]

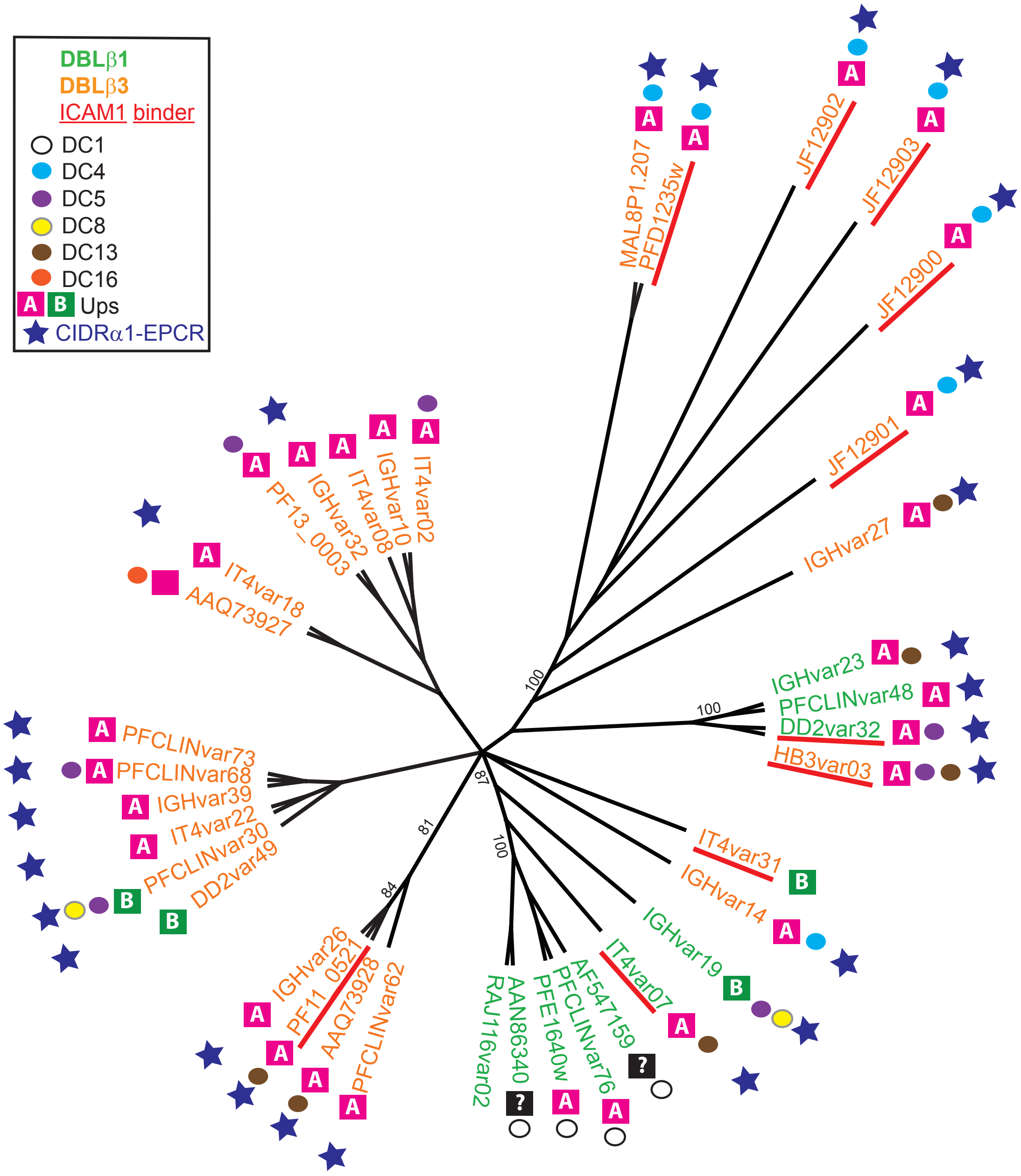

FIG. S2

Phylogeny of DBLβ1 and DBLβ3 domains. A neighbor joining cladogram of 38 DBLβ1/β3 domains that are located immediately after the PfEMP1 head structure. PfEMP1 encoding EPCR binding CIDR domain subtypes are labeled with an asterisk. Recombinant DBLβ1 or DBLβ3 domains that have been shown to bind ICAM-1 are underlined in red font. The Ups group that each gene belongs to is indicated by the letter within a box and types of domains cassettes present in each protein are indicated by ovals. The edge number by the origin of a branch indicates the bootstrap values (over 80%).
